# Hemodynamic Changes After Left Ventricular Assist Device Implantation Among Heart Failure Patients With and Without Elevated Pulmonary Vascular Resistance

**DOI:** 10.3389/fcvm.2022.875204

**Published:** 2022-04-26

**Authors:** Avishay Grupper, Israel Mazin, Kobi Faierstein, Adam Kurnick, Elad Maor, Dan Elian, Israel M. Barbash, Victor Guetta, Ehud Regev, Avi Morgan, Amit Segev, Jacob Lavee, Paul Fefer

**Affiliations:** ^1^Division of Cardiology, Leviev Center of Cardiovascular Medicine, Sheba Medical Center in Tel-Ha’Shomer, Ramat Gan, Israel; ^2^Sackler School of Medicine, Tel-Aviv University, Te-Aviv, Israel; ^3^Internal Medicine Department, Sheba Medical Center in Tel-Ha’Shomer, Ramat Gan, Israel; ^4^Department of Medicine, State University of New York, Downstate Health Sciences University, Brooklyn, NY, United States

**Keywords:** pulmonary vascular resistance, pulmonary hypertension, non-reversible, cardiac output, heart transplantation

## Abstract

**Background:**

Left ventricular assist devices (LVADs) may reverse elevated pulmonary vascular resistance (PVR) which is associated with worse prognosis in heart failure (HF) patients. We aim to describe the temporal changes in hemodynamic parameters before and after LVAD implantation among patients with or without elevated PVR.

**Methods:**

HF patients who received continuous-flow LVAD (HeartMate 2&3) at a tertiary medical center and underwent right heart catheterization with PVR reversibility study before and after LVAD surgery. Patients were divided into 3 groups: normal PVR (<4WU); reversible PVR (initial PVR ≥4WU with positive reversibility); and non-reversible (persistent PVR ≥4WU).

**Results:**

Overall, 85 LVAD patients with a mean age of 58 years (IQR 49–64), 65 patients (76%) were male; 60 patients had normal PVR, 20 patients with reversible and 5 patients with non-reversible PVR pre-LVAD. All patients with elevated PVR (≥4WU) had higher pulmonary pressures (PP) and increased trans-pulmonary gradient (TPG) compared to patients with normal PVR (*p* < 0.05). Patients with non-reversible PVR were more likely to have a significantly lower baseline cardiac output (CO) compared to all other groups (*p* ≤ 0.02). Hemodynamic parameters and PVR post LVAD were similar in all study groups. Patients with baseline elevated PVR (reversible and non-reversible) demonstrated a significant improvement in PP and TPG compared to patients with normal baseline PVR (*p* ≤ 0.05). The improvement in CO and PVR post-LVAD in the non-reversible PVR group was significantly greater compared to all other groups (*p* < 0.01). There were no significant differences between study groups in post LVAD and post heart transplantation course.

**Conclusion:**

Hemodynamic parameters improved after LVAD implantation, regardless of baseline PVR and reversibility, and enabled heart transplantation in patients who were ineligible due to non-reversible elevated PVR. Our findings suggest that mitigation of elevated non-reversible PVR is related to reduction in PP and increase in CO.

## Introduction

In patients with left ventricular heart failure (HF), the development of pulmonary hypertension (PH) is frequent and has significant impact on disease progression, morbidity, and mortality (1–5). The progression of PH among HF patients occurs in a stepwise fashion, starting with a gradual increase in left-sided filling pressures that can cause passive elevation of pulmonary venous and pulmonary arterial pressures. If untreated, long-standing PH can progress and cause pulmonary arterial vasoconstriction and remodeling with endothelial thickening, arteriolar smooth muscle hypertrophy, and fibrosis of the pulmonary vasculature, resulting in elevated pulmonary vascular resistance (PVR) (1–4). Elevated PVR has traditionally been associated with increased risk of early graft dysfunction and a worse prognosis post heart transplantation (HT) (6–9). The International Society of Heart Lung Transplant database consistently demonstrates a linear relationship between PVR and mortality after HT leading to gradual decrease in the threshold of pharmacologic irreversible PVR define as a contraindication for HT from 6WU to 4WU (10). Recent studies validated the association between PVR ≤4WU and better survival post HT (11). Thus PVR is among the key measurements derived from right heart catheterization (RHC) in the assessment of patients who are HT candidates (10, 12).

Left ventricular assist devices (LVADs) are increasingly utilized for advanced HF and have been used in patients with irreversible elevation of PVR. Reduction in PVR following LVAD implantation has been reported in previous studies that included small cohorts of patients with fixed elevated PVR, and mainly focused on the improvement in pulmonary pressures and PVR after LVAD implantation (13–17). Based on these studies, implantation of LVAD may be considered as a strategy for patients who are not HT candidates due to irreversible elevation of PVR, allowing subsequent re-evaluation to establish HT candidacy following implantation (13–17).

The mechanisms by which LVAD implantation results in reversal of irreversible elevated PVR are unknown at present. It might be speculated that LVADs induce reverse remodeling of the pulmonary vascular tree by continuously unloading the left ventricle and relieving pulmonary congestion. However, data regarding changes in hemodynamic parameters post LVAD in patients with or without elevated PVR before device implantation are scarce. Understanding these differences could guide the management of patients with persistently elevated PVR as potential candidates for LVAD implantation and subsequent evaluation for HT.

The aim of this study was to describe the temporal changes in hemodynamic parameters obtained from RHC following LVAD implantation among patients with or without elevated PVR, and to identify trends that may explain the improvement in reversible and non-reversible PVR post-LVAD.

## Materials and Methods

An observational study including all LVAD patients who received continuous-flow LVAD (HeartMate 2&3) at a tertiary medical center and underwent invasive RHC prior to LVAD implantation surgery and during their follow up at the LVAD clinic. For patients who had more than one RHC study, the last pre-LVAD RHC and the first post-LVAD RHC implantation were used. All patients underwent RHC *via* the right femoral vein using a 7 French Swan-Ganz catheter, and standard hemodynamic assessments were performed. All hemodynamic parameters were obtained at end expiration and reviewed by a HF cardiologist. Pulmonary capillary wedge pressure (PCWP) was measured as a mean of the a-wave pressure waveform. The measurement was based on the best waveform (the one with the minimum number of artifacts). To categorize patients, a threshold baseline pulmonary vascular resistance (PVR) value of 4 Wood units (WU) was applied. Reversibility of PVR was evaluated during RHC pre-LVAD by using intravenous sodium nitroprusside or inhaled nitric oxide for all patients with initial PVR ≥4WU. Reversible PVR was defined as post-medication PVR <4 WU. Patients were divided into 3 groups: normal PVR (<4WU); reversible PVR (initial PVR ≥4WU with positive reversibility; and non-reversible (fixed non-reversible PVR ≥4WU). All patient data were abstracted from the computerized medical record and included patient characteristics, echocardiographic and hemodynamic parameters at baseline and post-LVAD surgery. The local Institutional Review Board approved this retrospective analysis based on strict maintenance of participants’ anonymity during database analyses.

### Statistical Analysis

Our data do not follow a normal distribution pattern (Kolmogorov–Smirnov test and Shapiro-Wilk test), thus we used non-parametrical tests for statistical analyses. The chi-square or Fisher exact test were used for comparison of categorical variables. For continuous variables we used the Mann-Whitney U test or Kruskal-Wallis test which was appropriate. We further used the Wilcoxon signed-rank test to compare paired RHC measurements of pre and post LVAD implantation. Since our focus was the absolute numerical (or calculated) change between the RHC measurements, we used one-sided tests on the expected direction. For example, we expected cardiac output (CO) to increase post LVAD, hence this direction was calculated. All statistical analyses were performed using the R programming language, with *p* < 0.05 considered significant.

## Results

The study cohort included 85 LVAD patients with a mean age of 58 years (IQR 49–64), 65 (76%) of whom were male. Overall, based on pre-LVAD RHC, 60 patients had normal baseline PVR, 20 patients had reversible PVR, and 5 patients had irreversible PVR. Patient baseline characteristics are shown in Table 1. Patients with elevated baseline PVR (reversible and non-reversible) were more likely to present with chronic kidney disease compared to patients with normal PVR (36% vs. 8%, *p* = 0.0018). Other baseline characteristics did not differ between the 3 groups. There was also no significant difference in duration of HF disease between the study groups.

**TABLE 1:** Baseline patient characteristics.

	Overall *N* = 85	Normal *N* = 60	Reversible *N* = 20	Non-Reversible *N* = 5	p
Age at LVAD implant mean (SD)	58.00 [49.00, 64.00]	57.00 [49.00, 63.00]	61.50 [57.50, 64.50]	42.00 [41.00, 57.00]	0.052
Diabetes mellitus (%)	25 (30.1)	21 (36.2)	3 (15.0)	1 (20.0)	0.179
Hypertension (%)	31 (37.3)	18 (31.0)	11 (55.0)	2 (40.0)	0.160
Chronic renal failure (%)	14 (16.9)	5 (8.6)	7 (35.0)	2 (40.0)	0.009
Hyperlipidemia (%)	44 (53.0)	27 (46.6)	14 (70.0)	3 (60.0)	0.184
COPD (%)	26 (31.3)	21 (36.2)	5 (25.0)	0 (0.0)	0.193
Prior CABG (%)	5 (6.1)	3 (5.2)	2 (10.5)	0 (0.0)	0.588
Prior IHD (%)	45 (54.2)	29 (50.0)	13 (65.0)	3 (60.0)	0.492
Beta blockers (%)	57 (68.7)	40 (69.0)	15 (75.0)	2 (40.0)	0.319
ACE or ARB (%)	53 (63.9)	37 (63.8)	14 (70.0)	2 (40.0)	0.458
Statins (%)	31 (37.3)	24 (41.4)	6 (30.0)	1 (20.0)	0.471
MRA (%)	12 (14.5)	9 (15.5)	2 (10.0)	1 (20.0)	0.78
IV Furosemide (%)	18 (22.2)	14 (24.6)	2 (10.5)	2 (40.0)	0.273
CRT (%)					0.888
Non	52 (62.7)	36 (62.1)	12 (60.0)	4 (80.0)	
CRT-D	30 (36.1)	21 (36.2)	8 (40.0)	1 (20.0)	
CRT- P	1 (1.2)	1 (1.7)	0 (0.0)	0 (0.0)	
ICD (%)	22 (26.5)	15 (25.9)	5 (25.0)	2 (40.0)	0.778

*LVAD-left ventricular assist device; COPD- chronic obstructive pulmonary disease; CABG- coronary artery bypass graft surgery; IHD- ischemic heart disease; ACE- angiotensin converting enzyme; ARB- angiotensin receptor blocker; MRA- mineralocorticoid receptor antagonist; IV- intravenous; CRT- cardiac resynchronization therapy; CRT-D- cardiac resynchronization therapy with a defibrillator; CRT-P- cardiac resynchronization therapy without a defibrillator; ICD- implantable cardioverter-defibrillator.*

Echocardiographic parameters before and after LVAD implantation surgery are shown in Table 2. Patients with irreversible PVR were more likely to have a lower baseline left ventricular ejection fraction (LVEF) compared to other groups (*p* = 0.014). No other major differences in echocardiographic parameters were demonstrated between groups.

**TABLE 2:** Echocardiographic parameters before and after LVAD implantation.

	Overall *N* = 85	Normal *N* = 60	Reversible *N* = 20	Non-Reversible *N* = 5	p
**Baseline**					
LVEF% (median [IQR])	20.00 [10.00, 20.00]	15.00 [10.00, 20.00]	20.00 [15.00, 25.00]	10.00 [10.00, 13.00]	0.014
LVEDD (cm, median [IQR])	6.80 [6.20, 7.40]	6.80 [6.27, 7.40]	6.40 [5.60, 6.93]	7.30 [6.70, 7.30]	0.087
LVESD (cm, median [IQR])	5.70 [5.20, 6.70]	5.70 [5.30, 6.73]	5.30 [4.77, 6.12]	6.40 [6.20, 6.60]	0.093
LA Dimension (cm, median [IQR])	4.90 [4.60, 5.30]	4.90 [4.60, 5.40]	4.80 [4.50, 5.20]	4.90 [4.60, 5.20]	0.67
LA Area (cm^2^, median [IQR])	29.75 [25.55, 34.00]	30.00 [25.60, 34.80]	29.40 [24.15, 31.75]	26.00 [26.00, 33.00]	0.699
MR Degree (%)					0.485
Non	6 (7.1)	6 (10.2)	0 (0.0)	0 (0.0)	
Mild	26 (31.0)	19 (32.2)	7 (35.0)	0 (0.0)	
Moderate	38 (45.2)	24 (40.7)	11 (55.0)	3 (60.0)	
Moderate-Severe	6 (7.1)	4 (6.8)	1 (5.0)	1 (20.0)	
Severe	8 (9.5)	6 (10.2)	1 (5.0)	1 (20.0)	
TR Degree (%)					0.832
Non	15 (17.9)	11 (18.6)	4 (20.0)	0 (0.0)	
Mild	35 (41.7)	24 (40.7)	8 (40.0)	3 (60.0)	
Moderate	25 (29.8)	16 (27.1)	7 (35.0)	2 (40.0)	
Moderate-Severe	4 (4.8)	3 (5.1)	1 (5.0)	0 (0.0)	
Severe	5 (6.0)	5 (8.5)	0 (0.0)	0 (0.0)	
Enlarged RV (%)	35 (46.7)	21 (39.6)	11 (64.7)	3 (60.0)	0.162
RV function (%)					0.142
Non	15 (20.3)	13 (25.5)	2 (11.1)	0 (0.0)	
Mild	48 (64.9)	30 (58.8)	15 (83.3)	3 (60.0)	
≥Moderate	11 (14.9)	8 (15.7)	1 (5.6)	2 (40.0)	
SPAP, mmHg (mean (SD))	53.00 [47.00, 60.00]	53.00 [45.00, 57.00]	58.00 [52.25, 65.50]	54.50 [46.25, 57.25]	0.123
Post LVAD Implantation					
LVEDD (cm, median [IQR])	5.40 [4.43, 6.10]	5.40 [4.60, 6.40]	4.80 [4.25, 5.55]	5.90 [5.60, 6.30]	0.212
LVESD (cm, median [IQR])	4.30 [3.23, 5.38]	4.50 [3.35, 5.80]	3.45 [3.05, 4.32]	4.55 [3.55, 5.05]	0.098
LA dimensions (cm, median [IQR])	4.60 [4.20, 5.20]	4.60 [4.10, 5.20]	4.80 [4.40, 5.30]	4.50 [3.70, 5.20]	0.798
LA area (cm^2^, median [IQR])	25.20 [20.00, 28.00]	25.20 [20.00, 27.50]	24.00 [18.75, 27.50]	24.00 [22.00, 26.00]	0.991
MR degree (%)					0.564
Non	19 (38.8)	14 (37.8)	4 (50.0)	1 (25.0)	
Mild	20 (40.8)	17 (45.9)	1 (12.5)	2 (50.0)	
Moderate	9 (18.4)	5 (13.5)	3 (37.5)	1 (25.0)	
Moderate-Severe	1 (2.0)	1 (2.7)	0 (0.0)	0 (0.0)	
TR degree (%)					0.811
Non	26 (44.1)	19 (44.2)	6 (50.0)	1 (25.0)	
Mild	23 (39.0)	18 (41.9)	3 (25.0)	2 (50.0)	
Moderate	9 (15.3)	5 (11.6)	3 (25.0)	1 (25.0)	
Moderate-Severe	1 (1.7)	1 (2.3)	0 (0.0)	0 (0.0)	
Enlarged RV (%)	31 (58.5)	22 (59.5)	7 (50.0)	2 (100.0)	0.356
RV function (%)					0.369
Non	15 (24.2)	12 (26.7)	3 (20.0)	0 (0.0)	
Mild	29 (46.8)	18 (40.0)	10 (66.7)	1 (50.0)	
≥Moderate	18 (29.0)	15 (33.3)	2 (13.3)	1 (50.0)	

*LVEF- left ventricular ejection fraction; IQR- interquartile range; SD- standard deviation; LVEDD- left ventricular end diastolic diameter; LVESD- left ventricular end systolic diameter; LA- left atrium; MR- mitral regurgitation; TR- tricuspid regurgitation; RV- right ventricle; SPAP- systolic pulmonary arterial pressure.*

Baseline RHC was performed 18 days (IQR 5.75–48.75) before LVAD implantation surgery without difference in mean time from RHC to LVAD surgery between groups (*p* = 0.35). Table 3 shows RHC data before and after LVAD implantation. Patients in the non-reversible group demonstrated significantly lower cardiac output (CO) and cardiac index (CI) pre-LVAD compared to all other groups (*p* ≤ 0.02 for all). All patients with initial elevated PVR (≥4WU) had higher pulmonary pressures and increased trans-pulmonary gradient (TPG) compared to patients with normal PVR (*p* < 0.05). Patients in the non-reversible group had significantly higher PVR compared to all other patients (*p* < 0.001).

**TABLE 3:** Hemodynamic parameters before and after LVAD implantation.

	Overall *N* = 85	Normal *N* = 60	Reversible *N* = 20	Non-Reversible *N* = 5	*P*-Value
**Baseline**					
Days from RHC to LVAD (median [IQR])	18.00 [5.75, 48.75]	16.00 [5.50, 52.50]	27.00 [9.00, 41.00]	7.00 [3.00, 22.00]	0.349
Cardiac output, l/min (median [IQR])	3.04 [2.49, 3.84]	3.01 [2.50, 3.95]	3.21 [2.73, 3.66]	1.92 [1.43, 2.32]	0.02
Cardiac index, l/min/m^2^ (median [IQR])	1.60 [1.37, 2.00]	1.60 [1.40, 2.00]	1.75 [1.37, 1.92]	1.10 [0.90, 1.20]	0.019
PCWP, mmHg (median [IQR])	26.50 [19.75, 31.00]	25.00 [19.00, 31.00]	28.00 [22.75, 35.50]	26.00 [25.00, 26.00]	0.37
SPAP, mmHg (median [IQR])	57.50 [44.75, 68.25]	52.00 [42.00, 63.00]	68.50 [60.25, 84.25]	71.00 [68.00, 81.00]	0.001
DPAP, mmHg (median [IQR])	24.50 [18.00, 31.00]	23.00 [17.50, 29.50]	26.50 [23.00, 35.25]	39.00 [27.00, 40.00]	0.017
mPAP, mmHg (median [IQR])	38.00 [29.50, 43.50]	35.00 [26.50, 42.00]	43.00 [36.75, 48.50]	43.00 [42.00, 51.00]	0.005
Trans-pulmonary gradient (median [IQR])	10.00 [7.00, 14.00]	8.00 [6.00, 12.00]	15.00 [10.75, 17.25]	16.00 [11.00, 18.00]	<0.001
RApressure, mmHg (median [IQR])	7.00 [2.00, 12.00]	9.8 [8.00, 11.50]	10.3 [1.25, 12.00]	10.50 [2.25, 19.50]	0.811
PVR, WU (median [IQR])	3.08 [2.01, 4.88]	2.60 [1.86, 3.42]	4.90 [3.86, 6.28]	6.59 [6.42, 7.06]	<0.001
PVR, WU – Post medicine (median [IQR])			2.54 [2.15, 3.15]	6.48 [6.36, 6.81]	0.001
Trans-pulmonary vascular resistance (median [IQR])	26.50 [19.75, 31.00]	25.00 [19.00, 31.00]	28.00 [22.75, 35.50]	26.00 [25.00, 26.00]	0.37
Diastolic pulmonary pressure gradient (median [IQR])	−1.00 [−3.75, 2.00]	−1.00 [−4.00, 0.00]	−0.50 [−2.25, 3.00]	4.00 [2.00, 13.00]	0.071
Post LVAD Implantation					
Days from LVAD to RHC (median [IQR])	198.00 [133.00, 389.00]	236.00 [148.75, 425.00]	160.50 [108.75, 232.00]	207.00 [152.00, 210.00]	0.109
Cardiac output, l/min (median [IQR])	4.20 [3.58, 4.73]	4.21 [3.58, 4.90]	4.01 [3.58, 4.52]	4.63 [4.20, 4.87]	0.569
Cardiac index, l/min/m^2^ (median [IQR])	2.10 [1.90, 2.50]	2.10 [1.90, 2.50]	2.05 [1.90, 2.42]	2.70 [2.20, 2.90]	0.492
PCWP, mmHg (median [IQR])	12.00 [9.00, 18.00]	12.00 [8.75, 18.00]	13.50 [9.75, 19.25]	12.00 [9.00, 14.00]	0.84
SPAP, mmHg (median [IQR])	34.00 [27.00, 41.00]	32.50 [27.00, 41.00]	38.50 [30.75, 47.50]	45.00 [29.00, 47.00]	0.152
DPAP, mmHg (median [IQR])	16.00 [12.00, 20.00]	15.00 [11.75, 20.00]	18.00 [13.75, 20.00]	15.00 [14.00, 20.00]	0.408
mPAP, mmHg (median [IQR])	23.00 [18.00, 28.00]	21.00 [17.75, 27.00]	24.00 [19.50, 30.00]	27.00 [19.00, 31.00]	0.358
Trans-pulmonary gradient (median [IQR])	9.00 [7.00, 11.00]	9.00 [7.00, 10.00]	10.00 [9.00, 12.00]	10.00 [8.00, 15.00]	0.098
RA pressure (median [IQR])	11.00 [7.00, 17.00]	10.50 [6.75, 17.00]	12.50 [7.75, 17.00]	10.00 [7.00, 10.00]	0.576
PVR, WU (median [IQR])	2.17 [1.70, 2.94]	2.08 [1.55, 2.77]	2.43 [1.82, 2.85]	2.18 [1.93, 3.25]	0.333
Trans-pulmonary vascular resistance (median [IQR])	4.20 [3.58, 4.73]	4.21 [3.58, 4.90]	4.01 [3.58, 4.52]	4.63 [4.20, 4.87]	0.569
Diastolic pulmonary pressure gradient (median [IQR])	3.00 [1.00, 5.00]	2.00 [0.00, 4.00]	3.50 [1.75, 5.25]	2.00 [2.00, 6.00]	0.279

*RHC- right heart catheterization; LVAD- left ventricular assist device; IQR- interquartile range; PCWP- pulmonary capillary wedge pressure; SPAP- systolic pulmonary arterial pressure; DPAP- diastolic pulmonary arterial pressure; mPAP- mean pulmonary arterial pressure; RA- right atrium; PVR- pulmonary vascular resistance; WU- wood unit.s*

Post LVAD RHC was performed at 198 days (IQR 133–389) after LVAD implantation surgery without difference in mean time from LVAD implantation to between groups (*p* = 0.11). No significant differences were demonstrated in all hemodynamic parameters post-LVAD between study groups (Table 3). All patients showed improvement in CO, CI and lower filling pressures in cardiac chambers and pulmonary vasculature after LVAD implantation surgery. Specifically, overall PVR post LVAD was 2.17 WU (IQR 1.7–2.94) without significant differences between study groups (*p* = 0.33).

**FIGURE 1:**
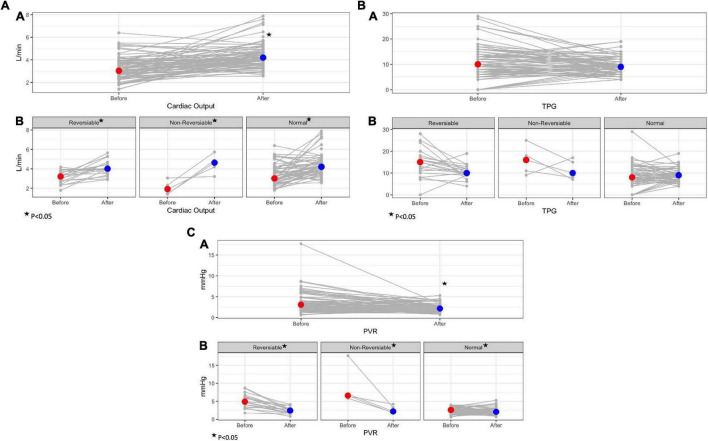
Trends in hemodynamic parameters before and after LVAD implantation surgery according to baseline PVR. **(A)** Trends in cardiac output before and after LVAD; A- overall study cohort; B- study groups according to baseline PVR. **(B)** Trends in total pulmonary gradient (TPG) before and after LVAD; A- overall study cohort; B- study groups according to baseline PVR. **(C)** Trends in pulmonary vascular resistance (PVR) before and after LVAD; A- overall study cohort; B- study groups according to baseline PVR.

To better appreciate the trends in parameters before and after LVAD implantation, a further analysis was made including the absolute difference in each parameter at baseline and post -LVAD for each patient group. Overall, all study groups reduced PCWP to a similar degree without significant differences among groups (*p* = 0.43). Patients with baseline elevated PVR (reversible and non-reversible) demonstrated a greater absolute improvement in pulmonary pressures and TPG compared to patients with normal baseline PVR (*p* ≤ 0.05). The improvement in CO and CI post-LVAD in the non-reversible group was significantly greater compared to all other groups (*p* = 0.016 and 0.017, respectively), hence, PVR post-LVAD improved remarkably in the non-reversible group compared to other groups (*p* < 0.01). Figure 1 demonstrates the changes in hemodynamic parameters before and after LVAD surgery in all study groups.

During follow up 4 patients (80%) in the non-reversible group, 6 patients (30%) in the reversible group, and 16 patients (27%) in the normal baseline PVR group underwent successful heart transplantation. There were no significant differences in post-transplantation course. Also, no significant differences in LVAD complications during follow-up were demonstrated between study groups.

## Discussion

Our study demonstrates the changes in hemodynamic parameters before and after LVAD implantation among different HF patient groups with either normal or elevated (reversible or non-reversible) PVR at baseline. The main findings of the current analysis are that non-reversible PVR was associated with significantly reduced baseline CO compared to all other study groups, and that LVAD therapy improved all hemodynamic parameters regardless of initial PVR. Furthermore, our data suggest that the mechanism of PVR improvement post LVAD is driven not only by a reduction in pulmonary pressure, but also by a significant increase in CO.

Pulmonary hypertension is a common finding in patients with advanced HF as the increased left ventricular diastolic filling pressure leads to a passive increase in post-capillary (pulmonary venous) pressure in the pulmonary circulation. Pulmonary venous congestion is often associated with a reactive increase in PVR. With time, however, pulmonary vasoconstriction followed by arterial wall remodeling, characterized by medial hypertrophy and intimal fibrosis, lead to PH which is unresponsive to pulmonary vasodilator treatment (1–4). Non-responsive PVR is considered a contraindication for HT (10, 12) as these patients have a greatly increased risk of morbidity and mortality due to acute right ventricular failure of the transplanted heart in the immediate postoperative period, a time at which the normal donor right ventricle is subjected acutely to a marked increase in workload (4, 6).

The PVR calculation is derived from the hydraulic version of Ohm’s law as input pressure is represented by mean pulmonary arterial pressure (mPAP), output pressure is represented by mean pulmonary venous pressure, which is equivalent to the PCWP or left atrial pressure, and the total flow is represented by the cardiac output, thus: PVR = mPAP- mPCWP/CO or TPG/CO. Hence, reduction in calculated PVR may result from either reduced TPG (the numerator of PVR equation) or increased CO (the denominator of PVR equation).

Previous studies evaluating the effect of LVAD among patients with elevated PVR focused on the mechanical unloading of the left ventricle by the pump, and secondary improvement in PH as the main mechanism of PVR reduction after LVAD implantation (13–17). The study by Mikus et al. presented significant reduction in mPAP, TPG and PVR among 27 patients with fixed elevated PVR prior to pulsatile and continues flow LVAD implantation (16). Kutty et al. retrospectively evaluated 17 patients with secondary PH and elevated PVR who underwent centrifugal LVAD implantation with significant reduction in pulmonary pressures, TPG and PVR during 6 months period post LVAD surgery (17). Our results support this notion in a larger cohort of centrifugal LVAD patients. Moreover, we compared hemodynamic changes among patients with and without elevated baseline PVR and demonstrated higher baseline pulmonary pressure in HF patients with elevated PVR compared to patients with normal PVR, and more significant decrease in absolute pulmonary pressure post LVAD among patients with elevated PVR.

Nevertheless, while LVADs may significantly improve pulmonary arterial pressure, they also simultaneously reduce PCWP in a proportionate manner, maintaining the difference between these 2 parameters (TPG) without significant change before and after LVAD implantation. Our results demonstrate that while mPAP and PCWP were significantly reduced post-LVAD (overall reduction of 14 mmHg for each parameter post LVAD in the entire cohort), with the net result being unchanged TPG before and after LVAD (overall reduction of 1 mmHg in the entire study cohort). However, this phenomenon was more common among patients with normal baseline PVR while patients with baseline elevated PVR (reversible and non-reversible) demonstrated a greater absolute improvement in pulmonary pressures and TPG compared to the reduction in PCWP.

Conversely, improvement in CO, the denominator of the PVR equation, will exert a similar effect on post-LVAD PVR. This mechanism is often demonstrated in invasive hemodynamic studies testing PVR reactivity among HT candidates. A positive response can be defined based on reduction of pulmonary pressure and PCWP with concomitant increase in CO (18). Nevertheless, changes in CO were not included in most previous studies evaluating LVAD therapy in elevated PVR patients (13–17). Gulati et al. evaluated a cohort of LVAD patients with elevated baseline PVR from the INTERMACS registry and reported that a higher baseline CO was associated with lower post-LVAD PVR over time (19). Crawford et al. reviewed the UNOS HT database and identified patients with normal mPAP and elevated PVR before HT. These patients had worse hemodynamics, as evidenced by a lower mean CO and higher TPG compared to patients with normal mPAP and normal PVR, demonstrating a possible situation of increased PVR due to low CO in the setting of normal mPAP (20). Our data highlights the significant effect of CO on PVR calculation, especially among HF patients with elevated seemingly non-reversible PVR. Not only did these patients present with significantly lower CO, CI and lower LVEF at baseline compared to the other study groups (including patients with elevated reversible PVR), but they also had a substantial improvement in CO and CI post LVAD (increase of 3.31 l/min [2.97, 3.43] and 1.90 l/min/m^2^ [1.70, 1.93], respectively, *p* < 0.02) compared to patients with normal or reversible elevated PVR (increase of 0.82–0.86 l/min [0.06, 1.86] in CO and 0.4 l/min/m^2^ [0.00, 0.82] in CI, *p* > 0.05). These findings demonstrate that elevated non-reversible PVR was mainly driven by lower CO at baseline, and that the significant improvement in PVR post LVAD among this patient group was achieved mainly as a result of a substantial increase in CO.

### Study Limitations

This analysis has all the inherent limitations of a small-size, single-center, observational study, especially regarding patients with non-reversible PH. As such generalization of the results should be applied with caution before confirmation is available from larger population analyses. Although the data were collected prospectively, our study is limited by its retrospective design.

## Conclusion and Clinical Implications

Following LVAD implantation we demonstrate improvement in all hemodynamic parameters, including CO and PVR, regardless of baseline PVR and reversibility. Greater improvements were observed in patients with higher PVR, especially in the small group of patients with non-reversible PVR. These findings highlight the impact of low CO in PVR calculation along with PH and elevated TPG and suggest that the improvement in seemingly non-reversible elevated PVR following LVAD therapy occurs not only by reduction of pulmonary pressure but also by increase in CO. We urge clinicians to carefully evaluate hemodynamic parameters in HF patients and consider LVAD therapy even in elevated non-reversible PVR for potential subsequent HT candidacy. Larger studies are warranted to validate our findings.

## Data Availability Statement

The raw data supporting the conclusions of this article will be made available by the authors, without undue reservation.

## Ethics Statement

The studies involving human participants were reviewed and approved by Sheba Medical Center Institutional Review Board. Written informed consent for participation was not required for this study in accordance with the national legislation and the institutional requirements.

## Author Contributions

All authors listed have made a substantial, direct, and intellectual contribution to the work, and approved it for publication.

## Conflict of Interest

The authors declare that the research was conducted in the absence of any commercial or financial relationships that could be construed as a potential conflict of interest.

## Publisher’s Note

All claims expressed in this article are solely those of the authors and do not necessarily represent those of their affiliated organizations, or those of the publisher, the editors and the reviewers. Any product that may be evaluated in this article, or claim that may be made by its manufacturer, is not guaranteed or endorsed by the publisher.
